# Determinants of *Schistosoma mansoni* in Sanja health center, north West Ethiopia

**DOI:** 10.1186/s12889-018-5552-0

**Published:** 2018-05-11

**Authors:** Asrat Atsedeweyn Andargie, Agmas Sisay Abera

**Affiliations:** 1 Department of Epidemiology and Biostatistics, Institute of Public Health, College of Medicine and Health Sciences, University of Gondar, P.O.Box 196, Gondar, Ethiopia; 2 Department of Statistics, College of Natural and Computational Sciences, University of Gondar, Gondar, Ethiopia

**Keywords:** *Schistosoma mansoni*, Ordinal logistic regression, Risk factors

## Abstract

**Background:**

In developing countries, *Schistosoma mansoni* is one of the chronic but neglected tropical diseases. In sub-Saharan Africa, the disease affects over 250 million people with nearly 800 million are at risk. In Ethiopia, *Schistosoma mansoni* is one of the most prevalent parasitic diseases. The aim of this study was to determine the prevalence and identify the determinant factors of *Schistosoma mansoni*, in terms of some socio-demographic variables and risk factors.

**Methods:**

A cross-sectional parasitological survey was conducted at Sanja health center, northwest Ethiopia from June 1 to June 30, 2015. A total of 228 study participants were included in the study. The participants were selected using systematic random sampling technique. Stool specimens were collected and examined using Kato-Katz methods. Structural questionnaires were used to collect data on socio-demographic variables and risk factors by face to face interviews. The major risk factors and demographic determinants of the infection status of *Schistosoma mansoni* were identified by using descriptive and ordinal logistic regression techniques.

**Results:**

The overall prevalence of *Schistosoma mansoni* was 16.67% (95%CI: 11.83–21.51%). Covariates such as no habit of swimming in rivers has lower risk (AOR = 0.022: 95%CI: 0.011–0.764), no frequency of swimming in rivers (AOR = 0.022: 95%CI: 0.0024–0.207), and 1 to 2 frequency of swimming (OR = 0.302: 95%CI: 0.097–0.941), washing clothes in rivers (AOR = 0.194: 95%CI: 0.046–0.0.811) and bathing in the river (AOR = 0.09: 95%CI: 0.010–0.815) were the most important determinant factors (*P*-value < 0.5) of *Schistosoma mansoni* in Sanja health center.

**Conclusion:**

In this study**,** the prevalence of *Schistosoma mansoni* was found to be high. Swimming habits, frequency of swimming, washing clothes, and bathing in rivers were found to be significant predictors of *Schistosoma mansoni*. Provisions of a safe water supply in the area and health education about the transmission of the *Schistosoma mansoni* infection are required.

## Background

Schistosomiasis is one of the chronic but neglected tropical diseases that leads to chronic infection. Chronic infection can lead to some serious conditions which can be damaging for an individual. Of these, cognitive impairment, infertility, fatigue, and sometimes, bladder cancer could be contracted via chronic infections. Currently in the globe, Schistosomiasis is ranked second after malaria as the most devastating parasitic disease which infects a sizable amount of individuals worldwide. About 250 million people are infected and around 800 million individuals are at risk of being infected globally [1]. Of these, 85% of the cases are found in 41 countries in Africa [2, 3].

It is endemic in over 74 developing countries of the world, causing over 300, 000 deaths per year mainly in sub-Saharan Africa [4, 5] and leading to the loss of 1.53 million disability [6]. In African continent, the infection with *S. mansoni* and *Schistosoma Haematobium* are the major causes for intestinal and urinary Schistosomiasis, respectively. Sub- Saharan Africa holds the greater amount (80–85%) of schistosomiasis infected people [7] where *S. mansoni* and *Schistosoma Haematobium* are the main contributing species. In the region, over 390 million individuals are at risk of being infected with *S. mansoni*. Of those who are at risk, over 50 million are infected. Likewise, approximately 436 million people are at risk of being infected with *S. Haematobium*. From those who are at risk of S. haematobium, 112 million of them are infected [5].

Schistosomiasis is a weakening disease that is affecting poor rural communities in sub-Saharan Africa. These populations seek water for drinking, cooking, washing and bathing which unfortunately are schistosomiasis transmission sites [8, 9]). In Ethiopia and Eritrea, the population living under the risk of infection with *S. mansoni* was estimated to be 19 million [10].

Ethiopia is ranking second after Nigeria in terms of the burden caused by schistosomiasis and more than 22 million of the populations are requiring preventive chemotherapy for it [11, 12]. Moreover, ecological changes and population movements have created suitable habitats for the snails. An estimated 4 million people are infected by the schistosomiasis [11]. A study by [13] revealed that significant association was found between *S. mansoni* infection and time taken to cover the distance between home and river, swimming habit, practice of river crossing, knowledge about schistosomiasis and its vector (*P* < 0.05).

In the past, different strategies have been used to prevent this debilitating disease. The primary health care approach has been used by a number of countries in Africa and other endemic regions of the world. Development of irrigation schemes, dam construction for hydroelectric power, water conservation for different purposes, human behaviours such as swimming habits, improper waste disposal, use of river water for different purposes, and wide distribution of intermediate host are identified as the major contributing factors for the increased prevalence and wide distribution of schistosomiasis [14].

Over time, new transmission of foci is being discovered in different parts of Ethiopia. The establishment of water resource development projects such as dams and irrigation and migration of people from endemic areas to previously non-endemic ones are seem to be the reasons for the spreading of the disease to new localities [15]. Schistosomiasis is common in northern Ethiopia as compared to south and south west Ethiopia [16]. The major factor that affects the distribution of both Schistosoma species in Ethiopia appears to be Temperature. *S. mansoni* is mainly found at altitudes between 1200 and 1900 m above sea level [17].

In Sanja town, northwest Ethiopia, one out of three patients are infected with *S. mansoni* [18]. Different studies on Schistosoma have been done on school children in Sanja area by considering specific age groups with some explanatory variables [19, 18]. However, no studies have been conducted in the area by including all age groups to find out the determinant factors of *Schistosoma mansoni*.

The present study was conducted to determine the risk factors and prevalence of *Schistosoma mansoni* by considering all age groups with populations that come from different areas. Hence, the objective of the study was to determine the prevalence and identify determinant factors of *Schistosoma mansoni* in Sanja health center, northwest Ethiopia.

## Methods

### Study area, design, and period

The study was conducted in Sanja’s health center, Tach-Armachiho district, northwest Ethiopia. That particular area has altitudes ranging from 1900 to 2200 m above sea level. Sanja is the capital of Tach Armachiho district which is surrounded by the Maho Stream and Sanja River. This river and stream contribute a sizable source, if not a majority, of the *S. mansoni* infection in that region [19]. A cross-sectional parasitological survey was conducted from June 1 to June 30, 2015.

### Source population and study population

The population of Sanja town was almost evenly distributed among sexes in 2015. As a result, there were 3591 males and 3664 females which totaled 7255 inhabitants in the town. Annually, 13,390 patients planned to give stool samples in the health center; and among these in the month of June, 1228 patients were expected to give stool samples to the health center. The Sanja river and Maho stream serves as sources of water for laundry, bathing and other domestic and recreational purposes. Most of the town’s inhabitants had no toilet and excreted their feces in open field, particularly in groves and shades of trees near the two water bodies.

### Sample size and sampling procedures

The sample size (n) was determined using the statistical formula.

$n=\frac{Z^2p\left(1-p\right)}{d^2}$ [20, 21]), where *n* is sample size, Z is 95% confidence interval (1.96), *p* is expected prevalence and it is determined from a pilot survey that was taken from Sanja health center’s patients. Among 50 random patients that gave stool samples 12 of them had *Schistosoma mansoni*, so *p* is 24% and *d* is precision or margin of error (5%). Hence, the required sample size was computed to be as follows,

$$ {n}_0=\frac{Z^2p\left(1-p\right)}{d^2}=\frac{(1.96)^2\ast 0.24\ast 0.76}{(0.05)^2}=280. $$

The value of $\frac{n_0}{N}=\frac{280}{1228}=0.228$ which is greater than 5%. When the sample represents a significant (i.e. over 5%) proportion of the population, a finite population correction factor can be applied. This will reduce the sample size required. The formula for this is:

$n=\frac{n_0}{1+\frac{\left({n}_0-1\right)}{N}}=\frac{280}{1+\frac{\left(280-1\right)}{1228}}=228$, where *n*is adjusted sample size, *n*_0_ is original required sample size and N is monthly expected population. Hence, the required sample size was computed to be 228. Systematic random sampling technique was used to select participants. Stool samples were taken from every fifth patient. The starting point was selected by using simple random sampling technique among five patients in the study.

### Variables of the study

The dependent variable was the infection status of *Schistosoma mansoni* among patients in the Sanja health center. The intensity of infection due to *S. mansoni* had ordinal distribution and defined as negative, light, moderate and heavy. Intensity of infection was estimated from the number of eggs per gram of stool (epg) and according to the [22] criteria. The classes of intensity were categorized as light (epg < 100), moderate (epg between 101 and 400) and heavy (epg > 400) [14]. The choice of these variables was guided by different literatures as the determinant factors of *S. mansoni*. These categories of the independent variables were coded starting from zero to make it appropriate for further analysis using different statistical models.

### Operational definitions

Knowledge about *Schistosoma mansoni* was measured by asking two questions. If the participant answered both of the questions, then we categorized as knowledgeable, otherwise not.

### Data collection procedures (instrument, personnel)

Primary data for socio-demographic variables and possible risk factors of *S.Mansoni* were gathered by investigators from Sanja health center’s patients on the basis of face to face interviews by using structured questionnaire. Stool samples was used. The stool cups was cleaned and preserved in 10% formalin and later processed using formal ether concentration techniques. Kato- thick smear was prepared from each fresh stool sample for quantitative egg count of *S. mansoni* infection and identify the severity of the disease. This was done by a laboratory technician in Sanja health center.

### Data quality management

The data quality was controlled through training of data collectors, pre-testing the tools before the main data collection was started, and made daily supervision.

### Data processing and analysis

The data were entered and cleaned in to EPI-Info version 7 and exported to SPSS version 22 for analysis. The variables were described with frequencies and percentages. Univariable and multiple ordinal logistic regressions was fitted since the outcome variable is ordinal. The *p*-value and adjusted odds ratio with 95% confidence interval were used to determine the association between the dependent and independent variables.

### Statistical models

Ordinal Logistic Regression (OLR) model was used to identify determinants of *Schistosoma Mansioni’s* status. Based on parasite concentration *Schistosoma mansoni* status is categorized into three groups; heavy, moderate, light, and no infection. Since *Schistosoma mansoni’s* status is ordinal, an OLR model with proportional odds assumption [23] was used to identify predictors of severe, moderate and light *Schistosoma mansoni* status if the proportional odds assumption is satisfied. The proportional odds model is widely used in biomedical and epidemiological applications [24]. The ordinal logistic regression model is fitted to the observed responses using the Maximum Likelihood Approach. Let Y denote an ordinal response variable with C levels (1, 2, …, *C*), and let ${\boldsymbol{X}}_{\boldsymbol{l}}^{\prime }=\left({x}_{1\boldsymbol{l}},{x}_{2\boldsymbol{l}},\dots, {x}_{p\boldsymbol{l}}\right),\boldsymbol{l}=1,2,\dots, n$ be a vector of p explanatory variables (covariates) for the ***l***^*th*^ subject. Let *π*_*i*_(*x*_***l***_) = *pr* (*Y*_***l***_ ≤ *i*| *x*_***l***_) denote the conditional probability of a response equal to *i* given${\boldsymbol{X}}_{\boldsymbol{l}}^{\prime }$, where *i* = 1, 2, …, *c* − 1. Suppose that we have a sample of *n* independent observations, denoted by (*x*_***l***_, *y*_***l***_), ***l*** = 1, …, *n*. For notational simplicity, let *Y*_*i****l***_ denote binary indicator variables of the response for subject ***l***, such that *y*_*i****l***_ = 1 if *y*_***l***_ ≤ *i* and *y*_*i****l***_ = 0 otherwise (*i* = 1, …, *c*; ***l*** = 1, …, *n*). Then:

$$ {\pi}_i\left({x}_{\boldsymbol{l}}\right)= pr\ \left({Y}_{\boldsymbol{l}}\le i|{x}_{\boldsymbol{l}}\right)= pr\ \left({Y}_{i\boldsymbol{l}}=1|{x}_{\boldsymbol{l}}\right)=\frac{e^{\left({\alpha}_i-{\beta}_1{x}_{1\boldsymbol{l}}-\dots -{\beta}_p{x}_{p\boldsymbol{l}}\right)}}{1+{e}^{\left({\alpha}_i-{\beta}_1{x}_{1\boldsymbol{l}}-\dots -{\beta}_p{x}_{p\boldsymbol{l}}\right)}}=\frac{e^{\left({\alpha}_i-{\boldsymbol{x}}_l^{\prime}\beta \right)}}{1+{e}^{\left({\alpha}_i-{\boldsymbol{x}}_l^{\prime}\beta \right)}} $$

$$ i=1,2,\dots, c-1;l=1,2,\dots, n $$

where *β* is a vector of regression coefficients and *α*_*i*_ is the ***i***^*th*^intercept coefficient.

## Results

### Sociodemographic characteristics of patients

Table 1 presents basic descriptive information that summarizes the associations between the determinant factors and infection status of *S. mansoni*. A total of 228 patients that gave stool samples from Sanja health center were eligible for this study. The prevalence of *Schistosoma mansoni* in Sanja health center was 16.67% (95%CI: 11.83–21.51%). Among these, 83.3% were negative for *S.Mansoni*, 9.2% had light *S.Mansoni*, and 7.5% had moderate /heavy *S.Mansoni*.

**Table 1:** Frequencies (percentage distribution) of infection status of schstosoma mansoni within categories of explanatory variables

Variables	Category	Infection status of S.mansoni		
		Negative (%)	Light (%)	Moderate or Heavy (%)
Sex of patients	Female	51.1	28.6	23.5
	Male	48.9	71.4	76.5
Age of patients	0–19	49.5	61.9	70.6
	20–39	36.8	28.6	23.5
	> = 40	13.7	9.5	5.9
Marital status of patients	Single	63.2	47.6	70.6
	Others	36.8	52.4	29.4
Educational level of patients	Illiterate	48.7	28.6	47.1
	Primary	30.7	47.6	29.4
	Secondary & above	20.6	23.8	23.5
Resident of patients	Rural	37.9	42.9	23.5
	Urban	62.1	57.1	76.5
Knowledge about *S. mansoni*	Yes	24.2	28.6	35.3
	No	75.8	71.4	64.7
Source of water	River(others)	15.8	38.1	35.3
	Pipe	84.2	61.9	64.7
Swimming in the river	Yes	15.8	28.6	76.5
	No	84.2	71.4	23.5
Frequency of swimming in the river	No	83.7	57.1	17.6
	1–2 per week	10.5	23.8	29.4
	> = 3 times per week	5.8	19.0	52.9
Water contact habits during crossing the river	Yes	48.9	66.7	94.1
	No	51.1	33.3	5.9
Washing clothes in the river	Yes	42.1	90.5	94.1
	No	57.9	9.5	5.9
Bathing in the river	Yes	46.3	95.2	100
	No	53.7	4.8	0.0
Fishing in the river	Yes	2.6	4.8	11.8
	No	97.4	95.2	88.2
previously treated for S.mansoni	Yes	6.8	14.3	11.8
	No	93.2	85.7	88.2
Participated in irrigation	Yes	1.6	4.8	5.9
	No	98.4	95.2	94.1
*S. masoni*	Total	83.3	9.2	7.5

Out of those patients who were negative of *S. mansoni*, 51.1% were females. Of these, 28.6% of the females and 71.4% of the males were positive for light intensity of *S.Mansoni*. Similarly, 23.5% of the females and 76.5% of the males were positive for intensity of moderate/heavy *S.Mansoni*. This showed that the infection status of *S.Mansoni* varies by age, marital status, educational level, resident of patients, frequency of swimming in the river, water contact habits during crossing the river, participating in irrigation, purposes around the river, and treatment history.

#### Univariable ordinal logistic regression analysis

Seventeen variables were selected and 17 uni-variable ordinal logistic regression models were fitted to assess the relationship between infection status of *S. mansoni* and the explanatory variables. The results are shown in Table 2. The variables such as sex of patients, source of water for drinking and cooking, water contact habit, swimming habit, frequency of swimming, washing clothes in the river and bathing in the river were significant predictors.

**Table 2:** Unadjusted odds ratio estimate from uni-variable ordinal logistic regression of each explanatory variable

		Unadjusted OR	Sig.	95% CI (UOR)	
				L B	UB
Sex of patients	Female	0.34	0.006	0.16	0.74
	Male	1	–	–	–
Age of patients	0–19	2.34	0.191	0.65	8.40
	20–39	1.25	0.753	0.32	4.90
	> = 40	1	–	–	–
Marital status of patients	Single	0.84	0.637	0.42	1.71
	Others	1	–	–	–
Education of patients	Illiterate	0.67	0.397	0.27	1.68
	Primary	1.09	0.857	0.43	2.73
	Secondary and above	1	–	–	–
Occupation of patients	Employed	0.31	0.264	0.04	2.44
	Un employed	1	–	–	–
Religion of patients	Orthodox	0.49	0.575	0.04	5.92
	Muslim	1	–	–	–
Resident of patients	Rural	0.82	0.594	0.39	1.70
	Urban	1	–	–	–
Knowledge about S. mansoni	No	0.68	0.321	0.32	1.45
	Yes	1	–	–	–
Source of Water	Pipe	0.33	0.004	0.15	0.71
	River(others)	1	–	–	–
Swimming habit	No	0.16	0.000	0.08	0.34
	Yes	1	–	–	–
Frequency of swimming in the river	No	0.07	0.000	0.03	0.17
	1–2 times per week	0.37	0.041	0.13	1.05
	> = 3 times per week	1	–	–	–
Water contact habit during crossing the river	No	0.24	0.001	0.11	0.56
	Yes	1	–	–	–
Washing clothes in the river	No	0.06	0.000	0.02	0.21
	Yes	1	–	–	–
Bathing in the river	No	0.02	0.000	0.00	0.17
	Yes	1	–	–	–
Fishing in the river	No	0.29	0.086	0.07	1.19
	Yes	1	–	–	–
Previously treated for S. mansoni	No	0.50	0.214	0.17	1.49
	Yes	1	–	–	–
Participating in irrigation	No	0.29	0.176	0.05	1.73
	Yes	1	–	–	–

#### Multiple ordinal logistic regression analysis

Here, we considered two models: Model I contained all the 17 covariates while Model II excluded marital status, age, educational level, occupation, religion, resident, knowledge of *S. mansoni*, fishing in the river, treatment history and irrigation from the analysis. Ordinal logistic regression was assessed using deviance-based chi-square test. Accordingly, the deviance-based chi-square test provided a chi-square value of 71.843 (*p* < 0.001) for Model II and 91.582 (*p* = .001) for Model I which would implied that both models fitted the data adequately. In logistic regression, there are several possible ways to measure the difference between the observed and fitted values. In logistic regression, the difference between the observed and fitted values are calculated for each covariate pattern depending on the estimated probability for that covariate pattern. The hypothesis that the model adequately fits the data can then be examined by the Pearson and Deviance tests.

To choose the best fit model among these two, we can use the AIC criteria, BIC criteria and deviance test. The −2 log  − likelihood values for models I and II were160.796 and 100.848, respectively. The deviance test statistic was calculated as:

$$ \mathrm{D}=-2\left[\ln \left({\mathrm{L}}_{\mathrm{I}}\right)-\ln \left({\mathrm{L}}_{\mathrm{I}\mathrm{I}}\right)\right]=160.796-100.848=59.948 $$

The value of the Chi-square distribution with 12 degrees of freedom was 26.22 at 0.01 level of significance. Since the deviance test statistic was significant at 1% level, the Model II was a better fit. The two candidate models were also compared using AIC and BIC. AIC values for Model I and II were 200.796 and 116.848, while the BIC values were 207.955 and 119.711, respectively. Since Model II had smaller values of both AIC and BIC, it was a better fit to the data. The results of multiple ordinal logistic regressions for Model II are shown in Table 3.

**Table 3:** Adjusted odds ratio estimate from model II

		AOR	Sig.	95% CI (AOR)	
				L B	UB
Sex of patients	Female	0.439	0.090	0.879	5.906
	Male(ref.)	1	–	–	–
Source of Water for drinking and cooking	Pipe	0.687	0.395	0.613	3.456
	River/others (ref.)	1	–	–	–
Swimming habit	No	0.09	0.027	0.011	0.764
	Yes	1	–	–	–
Frequency of swimming in the river	No	0.022	0.001	0.0024	0.207
	1–2 times per week	0.302	0.039	0.097	0.941
	> = 3 times per week	1	–	–	–
Water contact during crossing the river	No	0.832	0.763	0.251	2.759
	Yes(ref.)	1	–	–	–
Washing clothes in the river	No	0.194	0.025	0.046	0.811
	Yes(ref.)	1	–	–	–
Bathing in the river	No	0.09	0.032	0.010	0.815
	Yes(ref.)	1	–	–	–

## Discussions

A cross-sectional parasitological survey involving 228 persons in the Sanja health center, northwest Ethiopia, was conducted from June 1 to June 30, 2015 to identify the risk factors of *S. mansoni* in the Sanja health center.

The multiple ordinal logistic regression analysis was employed to select the most important determinants of *S. mansoni*. The results displayed in Table 3 showed that swimming habits, frequency of swimming habits, washing clothes, and bathing in the river were found to be statistically significant predictors of *S. mansoni*. The results indicated that swimming habits is a significant covariate. The estimated odds ratio (AOR 0.09) indicated that patients who do not have swimming habit in the river were 0.09 times less likely to have moderate or heavy *S. mansoni* as compared to that of patients who have swimming habits in the river keeping all other covariates fixed. The odds ratio could be as minimum as 0.011 and as maximum as 0.76 with 95% confidence. The result is consistent with [18, 19] and [25–27] that swimming in the river showed significant effects on the occurrence of *S. mansoni*. Table 3 showed that frequency of swimming was found to be statistically significant with *S. mansoni*. The estimated odds ratio (AOR = 0.022) implied that patients who do not swim in the river were 0.022 times less likely to have moderate/heavy *S. mansoni* than those patients who swim three times and above per week in the river holding all other variables constant. The odds ratio could be as low as 0.0024 and as high as 0.207 with 95% confidence. Similarly, the estimated odds ratio (AOR = 0.302) implied that patients who swim once or twice per week were 0.302 times less likely to have moderate/ heavy *S. mansoni* than those patients who swim greater than or equal to three times per week in the river keeping all other covariates constant. The odds ratio could be as minimum as 0.097 and as maximum as 0.941 with 95% confidence. This study result is consistent with [26, 27] that frequency of water contact habits showed significant effects on *S. mansoni* infection.

The results also revealed that washing clothe is a significant covariate. The estimated odds ratio (AOR = 0.194) indicated that patients who do not wash clothes in the river were 0.194 times less likely to have moderate/ heavy *S. mansoni* than that of patients who wash clothes in the river holding all other variables constant. This figure can go up to 0.811 and can go down to 0.046 with 95% confidence. This result of the study agreed with that of [25, 19] that washing clothes in the river had significant effect on *S. mansoni*. The model results portrayed that bathing in the river is a significant variable. The estimated odds ratio (AOR = 0.09) implied that patients who do not bath in the river were 0.09 times less likely to have moderate/heavy *S. mansoni* than that of patients who bath in the river keeping all other covariates fixed. The odds ratio could be as low as 0.0099 and as high as 0.815 with 95% confidence. The result is consistent with [25, 18, 26, 19 27] that bathing in the river was the high risk activities for the occurance of *S. mansoni* infection.

### Conclusions and recommendations

The prevalence of *Schistosoma mansoni* was high in Sanja health center. The study also revealed that four covariates had significant effects on the infection status of *S. mansoni* at Sanja health center. The results of the proportional odds model showed that swimming habits in the river, frequency of swimming habits in the river, washing clothes in the river and bathing in the river were the most important determinant factors of *S. mansoni* in the Sanja health center. Specifically, the study revealed that a higher infection status of *S. mansoni* was more likely the case for those patients who have swimming habits in the river as compared to that of patients who do not have swimming habits in the river. Considering frequency of swimming habits in the river, patients who swim frequently in the river were at a higher risk of *S. mansoni* as compared to those patients who do not swim in the river. Patients who wash clothes in the river were also more vulnerable to *S. mansoni* than those patients who do not wash clothes in the river. The result revealed that a higher infection status of *S. mansoni* was more likely for those patients who bathe in the river as compared to those patients who do not bathe in the river.

Provision of safe water supply in the area, health education about the transmission of *S. mansoni* infection and its control and treating rivers with available mollusicides in the area are needed. Finally, further research needs to be taken by including other factors which are not included in the study (like time of swimming, distance of home to water bodies and sanitary facilities).

## Abbreviations

AIC: Akaike information criteria
AOR: Adjusted odds ratio
BIC: Bayesian information criteria
CI: Confidence interval
MoH: Ministry of health
NTD: Neglected tropical diseases
OLR: Ordinal logistic regression
OR: Odds ratio
PO: Proportional odds
POM: Proportional odds model
S.MANSONI: *Schistosoma mansoni*
UOR: Unadjusted odds ratio
WHO: World Health Organization

## References

[1] WHO (2017). Global, regional, and national age-sex specific mortality for 264 causes of death, 1980–2016: a systematic analysis for the global burden of disease study. Lancet.

[2] WHO (1991). Basic laboratory methods in medical parasitology.

[3] The End Fund. Ending neglected diseases. http://www.end.org/schistosomiasis. Accessed 2017.

[4] Cheesbrough M (2001). Medical laboratory manual for tropical countries.

[5] Van der Werf MJ, de Vlas SJ, Brooker S, Looman CW, Nagelkerke NJ, Habbema JD, Engels D (2003). Quantification of clinical morbidity associated with schistosome infection in sub-Saharan Africa. Acta Trop.

[6] Gryseels B, Polman K, Clerinx J, Kestens L (2006). Human schistosomiasis. Lancet.

[7] WHO. Schistosomiasis and soil transmitted helminth infections. Wkly Epidemiol Rec 2006; 81: 145–164. http://www.who.int/wer/2006/wer8116.pdf, Accessed 2018.

[8] King H, Blanton E, Muchiri M, Ouma H, Kariuki C, Mungai P, Magak P, Kadzo H, Ireri E, Koech K (2004). Low heritable component of risk for infection intensity and infection-associated disease in urinary schistosomiasis among Wadigo Village populations in Coast Province, Kenya. Am J Trop Med Hyg.

[9] Johnson J, Lund J, Hartson B, Yoshino P (2009). Community diversity reduces Schistosoma mansoni transmission, host pathology and human infection risk. Proc R Soc B.

[10] Tedela S, Jemaneh L (1998). Schistosomiasis in Ethiopia and Eretria.

[11] MoH. National Master Plan for Neglected Tropical Diseases (2013–2015). Addis Ababa, Ethiopia; 2013. https://ntdenvision.org/sites/default/files/docs/national_ntd_master_plan_ethiopia_2013-2015_1.pdf.

[12] WHO. Investing to Overcome the Global Impact of Neglected Tropical Diseases. *Third WHO report on neglected tropical diseases,* Geneva. 2015.

[13] Gebretsadik G, Abat A, Hailu T (2017). Epidemiological study on Schistosoma mansoni infection in Diza area, Benishangul-Gumuz region, Northwest Ethiopia. Int J Curr Res.

[14] WHO Expert Committee (2002). Prevention and control of schistosomiasis and soil-transmitted helminthiasis. World Health Organ Tech Rep Ser.

[15] Erko B, Gemetchu T, Gemeda N, Dessie S (1996). Transmission of intestinal schistosomiasis in Addis Ababa, Ethiopia. E Afr Med J.

[16] Haile G (1994). Intestinal parasitism among Jiren elementary and junior secondary students in south West Ethiopia. Ethiop J Health Dev.

[17] Erko B, Balcha F, Kifle D (2006). The ecology of Biomphalaria sudanica in Lake Ziway, Ethiopia. Afr J Ecol.

[18] Alebie G, Erko B, Aemero M, Petros B (2014). Epidemiological study on Schistosoma mansoni infection in Sanja area, Amhara region, Ethiopia. BMC Parasit Vectors.

[19] Daniel WW (1999). Biostatistics: a Foundation for Analysis in the health sciences.

[20] Lwanga SK, Lemeshow S (1991). Sample size determination in health studies: a practical manual.

[21] WHO (2008). The social context of schistosomiasis and its control: an introduction and annotated bibliography.

[22] McCullagh P (1980). Regression models for ordinal data with discussion. J R Stat Soc Ser B (Methodol).

[23] Ananth CV, Kleinbaum DG (1997). Regression models for ordinal responses: a review of methods and applications. Int J Epidemiol.

[24] Ligabaw Worku, Demekech Damte, Mengistu Endris, Habtie Tesfa, and Mulugeta Aemero. Schistosoma mansoni infection and associated determinant factors among school children in Sanja town, Northwest Ethiopia. J Parasitol Res 2014; doi.org/10.1155/2014/792536 10.1155/2014/792536 PMC3995176 24800058

[25] Dejenie T, Legese K, Tomas Z, Kiros S (2013). Index of potential contamination for intestinal schistosomiasis among school children of Raya Alamata district, northern Ethiopia. Momona Ethiopian J Sci.

[26] Assefa A, Dejenie T, Tomass Z (2013). Infection prevalence of Schistosoma mansoni and associated risk factors among school children in suburbs of Mekelle city, Tigray, northern Ethiopia. Momona Ethiopian J Sci.

[27] Yimer M, Abera B, Mulu W (2014). Soil transmitted helminths and Schistosoma mansoni infections in elementary school children at tach Armachiho district, north-West Ethiopia. J Appl Sci Res.

